# Métastases musculaires squelettique asymptomatique d'un cancer bronchique non à petites cellules

**DOI:** 10.11604/pamj.2015.22.181.7994

**Published:** 2015-10-22

**Authors:** Mohammed Raoufi, Mohamed Oukabli, Abdelhamid Biyi, Hanane Elouazzani, Ismail Abderrahman Rhorfi, Ahmed Abid

**Affiliations:** 1 Service de pneumologie, Hôpital Militaire Mohamed V, Rabat, Maroc; 2 Servie d'anatomopathologie, Hôpital Militaire Mohamed V, Rabat, Maroc; 3 Service de médecine nucléaire, Hôpital Militaire Mohamed V, Rabat, Maroc

**Keywords:** Métastases, muscle, squelettique, cancer, metastases, muscle, skeletal, cancer

## Abstract

Le cancer bronchique reste parmi les cancers les plus agressifs malgré les avancées diagnostiques et thérapeutiques, les métastases à distance constituent l’élément majeur d'un mauvais pronostic. Nous rapportons une observation de métastases musculaires chez un patient porteur d'un cancer du poumon inopérable. La détection de cette métastase était grâce au TEP scan au 18 FDG. Ce bilan a conduit à un traitement par chimiothérapie systémique après biopsie exérèse de la localisation fessière. Les métastases musculaires squelettiques du cancer bronchique sont rares mais bien qu'indiquant un mauvais pronostic, elles sont accessibles à un traitement local efficace.

## Introduction

Le cancer du poumon représente l'une des principales causes de décès par cancer. Malgré les progrès en matière de diagnostic et de traitement, la survie globale à 5 ans reste sombre à 14% pour tous les stades [1]. Les sites métastatiques à distance dans le cancer du poumon comprennent généralement les glandes surrénales, le foie, les os et le cerveau [2]. L'imagerie joue un rôle important dans la classification de la tumeur. La tomographie par émission de positrons (TEP) au (18 FDG) est maintenant la plus utilisée pour la caractérisation des nodules pulmonaires, la recherche des métastases, la stadification, la planification du traitement et l’évaluation de la réponse au traitement dans le cancer du poumon [3]. Nous rapportons un cas rare de métastases d'un carcinome bronchique au niveau du muscle grand fessier détecté par TEP SCAN.

## Patient et observation

**Figure 1:**
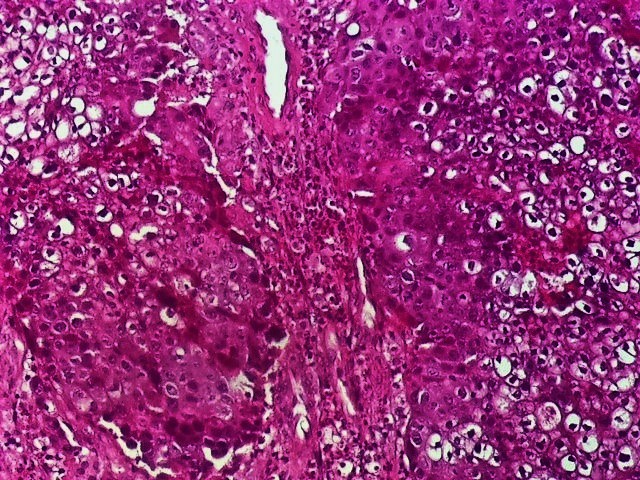
Infiltration pulmonaire par un carcinome à grandes cellules sans différenciation glandulaire ni épidermique visible (HEx40)

**Figure 2:**
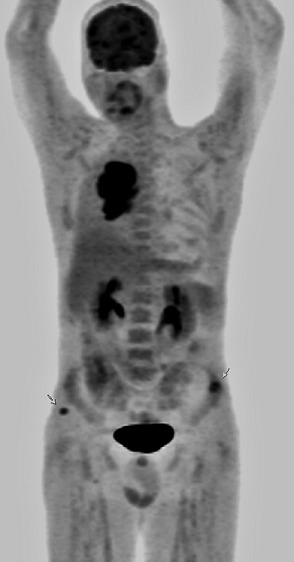
Pet scann montrant l'hypermétabolisme pathologique des trois sites pathologiques

**Figure 3:**
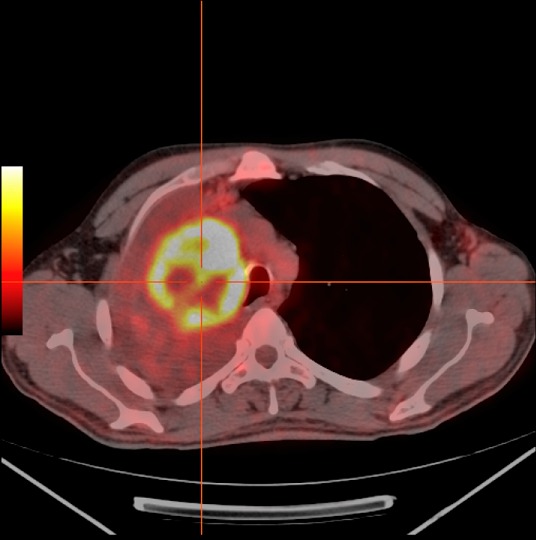
Hypermétabolisme (SUVmax bw: 9.9) sur le champ pulmonaire droit

**Figure 4:**
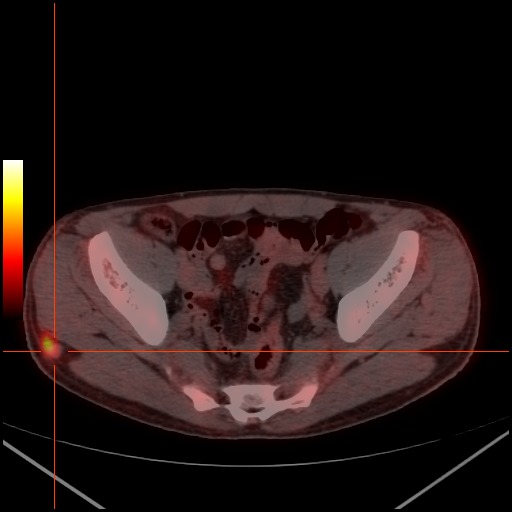
Foyer hyperméabolique (SUVmax bw: 4.3) dans la région fessière droite

**Figure 5:**
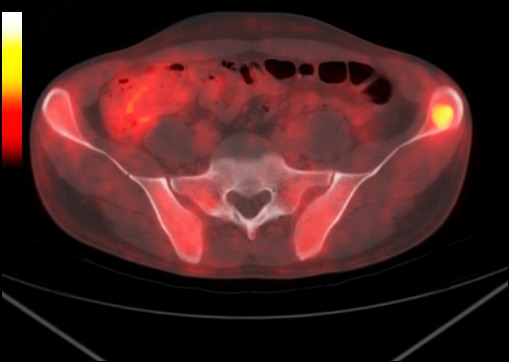
Foyer hyperméabolique (SUVmax bw: 4.1) touchant l'aile iliaque gauche

**Figure 6:**
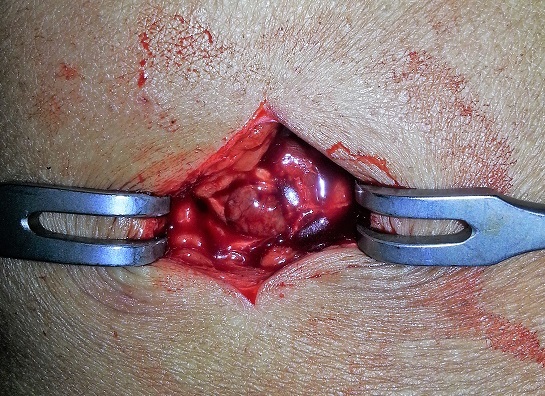
Exérèse de la tumeur fessière

**Figure 7:**
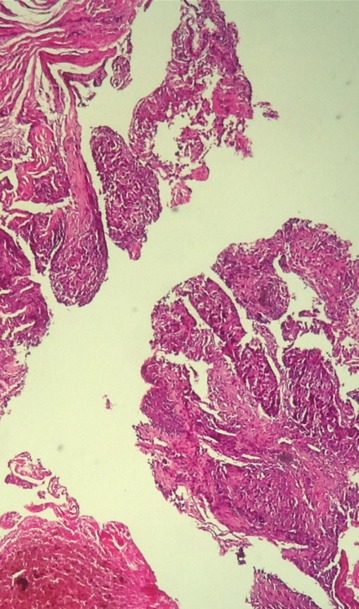
Infiltration de tissu conjonctivo-musculaire par le même processus à grandes cellules (HEx10)

Il s'agit d'un patient âgé de 45 ans, tabagique chronique admis pour dyspnée stade III (NYHA) associée à une toux sèche persistante. L'examen clinique objective un syndrome d’épanchement liquidien droit. Le scanner thoracique montre un processus lésionnel endo-bronchique obstruant la bronche souche droite qui bombe au niveau de la bifurcation bronchique, avec atélectasie du poumon droit associée à une adénopathie latéro-trachéale droite mesurant 52 x 47 mm et réaction pleurale du même coté. Le bilan biologique montre un taux d'hémoglobine à 13,8; une VS à 47mm; un ionogramme sanguin normal, la recherche de BK dans les expectorations est revenue négative. La fibroscopie bronchique a montré un bourgeon au niveau de la bronche souche droite dont la biopsie était en faveur d'un carcinome à grande cellules (Figure 1). La Spiromètrie objectivait un syndrome restrictif avec un VEMS à 42% de la valeur théorique, une CV à 35% de la valeur théorique, un rapport de Tiffneau à 98. Le bilan d'extension réalisé chez le patient montre, une TDM cérébrale normale, un TEP SCAN qui a objectivé 3 sites de fixations: une de la tumeur endothoracique, une au niveau de l’épine iliaque droite, et une en intramusculaire au niveau de la région fessière gauche (Figure 2, Figure 3, Figure 4, Figure 5). Une biopsie exérèse de la tumeur métastatique a été réalisée (Figure 6). Sa vérification histologique était en faveur d'une localisation secondaire d'un carcinome non à petites cellules d'origine pulmonaire (Figure 7). Le patient a été classé stade IV et traité par chimiothérapie antimitotique. Le patient est décédé deux ans après le début du traitement.

## Discussion

Le cancer du poumon est l'une des principales causes de décès par cancer chez les hommes et les femmes à travers le monde. La plupart des patients se présentent à un stade avancé, et donc malgré les progrès dans le diagnostic et le traitement, la mortalité reste élevée. Les métastases à distance du cancer du poumon comprennent généralement les glandes surrénales, les os et le cerveau. La tomodensitométrie thoracique avec des coupes au niveau de l'abdomen, une scintigraphie osseuse et l'imagerie cérébrale (TDM ou l'imagerie par résonance magnétique (IRM)) ont été considérés comme le bilan d'extension jusqu’à l'avènement de la TEP. Les métastases musculaires squelettiques du cancer du poumon sont rares avec très peu de cas décrits dans la littérature [4–6]. Divers facteurs physiologiques comme la circulation sanguine des tissus, la pression et le métabolisme ont été cités comme les raisons possibles des métastases musculaires squelettiques. Selon certaines études, la présence de certains inhibiteurs des protéases dans les tissus musculaires sont responsables du blocage de l'invasion et la croissance tumorale [7, 8]. Il a été montré que la TEP au FDG est une excellente méthode d'imagerie pour l’évaluation à l'exception des métastases cérébrales. Il peut détecter les métastases occultes dans environ 10-20% des cas de carcinome pulmonaire non à petites cellules (CBNPC) [9–11]. Dans une étude de 167 patients de CBNPC (stades I - III), la TEP-FDG a détecté des métastases dans une forte proportion de patients qui étaient autrement candidats pour un traitement à visée curative [12]. Cependant, sur les 32 cas de métastases à distance dans l’étude, il n'y avait aucun cas de métastase musculaire squelettique. La plupart des cas de métastases musculaires squelettiques décrites dans la littérature présentaient des symptômes cliniques à type de douleur et d'enflure au site affecté. Cependant, notre patient n'a eu aucun symptôme lié au site de métastases squelettiques musculaire. Il a été seulement détecté au PET-scan, qui a révélé l'absorption focale intense dans le muscle grand fessier. Bien que l'absorption de FDG focal dans le tissu sous-cutané profond de la région fessière soit assez souvent observée à la suite de l'inflammation au site d'injection, la nature de l'absorption intense et son emplacement dans le muscle profond était assez suspecte pour justifier une évaluation plus poussée. Une biopsie exérèse a confirmé la nature de la lésion métastatique fessière. L'absorption solitaire du FDG en extra-pulmonaire chez les patients atteints de cancer du poumon doit être prise en considération comme c'est le cas dans notre cas- puisque près de la moitié de ces lésions peut avoir une étiologie maligne [13]. L'utilisation de la TEP-TDM est un excellent bilan d'extension pour divers cancers, et qui a récemment donné lieu à plusieurs cas de métastases occultes à des endroits inhabituels. Les métastases inhabituelles de cancer du poumon comme celles du côlon et des muscles extra-oculaires ont été détectés par la TEP-TDM [14, 15]. Le traitement radical de la lésion métastatique solitaire est considéré comme une option de traitement, notre patient a été traité ensuite par chimiothérapie en vue de la nature systémique de la maladie. Ainsi la TEP TDM, en raison de toute sa capacité de dépistage, peut démasquer des sites métastatiques inhabituels lors de la présentation initiale et peut aider à réduire la chirurgie inappropriée chez ces patients.

## Conclusion

Les métastases musculaires squelettiques du cancer bronchiques sont rares mais non négligeables, elles sont souvent asymptomatiques, leur détection par un examen de haute performance change souvent l'attitude thérapeutique chez le patient.

## References

[1] Mountain CF (1997). Revisions in the international system for staging lung cancer. Chest..

[2] Pantel K, Izbicki J, Passlick B (1996). Frequency and prognostic significance of isolated tumour cells in bone marrow of patients with non-small cell lung cancer without overt metastases. Lancet..

[3] Mavi A, Lakhani P, Zhuang H, Gupta NC, Alavi A (2005). Fluorodeoxyglucose-PET in characterizing solitary pulmonary nodules, assessing pleural diseases, and the initial staging, restaging, therapy planning, and monitoring response of lung cancer. Radiol Clin North Am..

[4] Sridhar KS, Rao RK, Kunhardt B (1987). Skeletal muscle metastases from lung cancer. Cancer..

[5] McKeown PP, Conant P, Auerbach LE (1996). Squamous cell carcinoma of the lung: an unusual metastasis to pectoralis muscle. Ann Thorac Surg..

[6] Di Giorgio A, Sammartino P, Cardini CL (2004). Lung cancer and skeletal muscle metastases. Ann Thorac Surg..

[7] Eisenstein R, Kuettner KE, Neapolitan C (1975). The resistance of certain tissues to invasion. Am J Pathol..

[8] Sorgente N, Kuettner KE, Soble LW (1975). The resistance of certain tissues to invasion: II. Evidence for extractable factors in cartilage which inhibit invasion by vascularized mesenchyme. LabInvest..

[9] Earnest F, Ryu JH, Miller GM (1999). Suspected non-small cell lung cancer: incidence of occult brain and skeletal metastases and effectiveness of imaging for detection - pilot study. Radiology..

[10] Weder W, Schmid RA, Bruchhaus H (1998). Detection of extrathoracic metastases by positron emission tomography in lung cancer. Ann Thorac Surg..

[11] Pieterman RM, van Putten JWG, Meuzelaar JJ (2000). Staging of non-small-cell lung cancer with positron-emission tomography. N Engl J Med..

[12] MacManus MP, Hicks RJ, Matthews JP (2001). High rate of detection of unsuspected distant metastases by PET in apparent stage III non-small-cell lung cancer: implications for radical radiation therapy. Int J Radiat Oncol Biol Phys..

[13] Lardinois D, Weder W, Roudas M (2005). Etiology of solitary extrapulmonary positron emission tomography and computed tomography findings in patients with lung cancer. J Clin Oncol..

[14] Stinchcombe TE, Socinski MA, Gangarosa LM, Khandani AH (2006). Lung cancer presenting with a solitary colon metastasis detected on positron emission tomography scan. J Clin Oncol..

[15] Nguyen BD, Roarke MC (2008). Choroidal and extraocular muscle metastases from non-small-cell lung carcinoma: F-18 FDG PET/CT imaging. Clin Nucl Med..

